# The Effect of High-Quality RDX on the Safety and Mechanical Properties of Pressed PBX

**DOI:** 10.3390/ma15031185

**Published:** 2022-02-04

**Authors:** Shixiong Chen, Hua Qian, Bingxin Liu, Feiyang Xu, Jiuhou Rui, Dabin Liu

**Affiliations:** 1 School of Chemical Engineering, Nanjing University of Science and Technology, Nanjing 210094, China; shixiong_chen@126.com (S.C.); qianhua@njust.edu.cn (H.Q.); 18252501712@163.com (B.L.); iem_liu@163.com (F.X.); 2 State Key Laboratory of Explosion Science and Technology, Beijing Institute of Technology, Beijing 100081, China

**Keywords:** high-quality RDX, thermal decomposition performance, impact sensitivity, mechanical performance, compressive strength

## Abstract

In order to investigate and compare the effects of RDX crystal quality on the safety and mechanical properties of pressed PBX, different RDX-based PBXs were prepared by a water suspension granulation method. The surface morphology, thermal decomposition properties, impact sensitivity, and mechanical properties of high-quality RDX (H-RDX) and PBX were characterized by SEM, optical microscope, DSC, impact sensitivity tester, and universal material testing machines. The results have shown that the H-RDX crystal has a smoother surface, regular shape, higher density, fewer defects, better thermal stability, and lower impact sensitivity than raw RDX. The activation energy of H-RDX-based PBX is 26.0% higher than that of raw RDX-based PBX, and *H*_50_ increased by 2.8 cm, indicating that the application of H-RDX to PBX can effectively improve its thermal stability and reduce the impact sensitivity in the safety performance. However, the compressive strength of pressed H-RDX-based PBX is 36% lower than that of pressed raw RDX-based PBX, showing that H-RDX results in the deterioration of the compressive strength of pressed PBX in mechanical performance. Fortunately, this study found a strategy on how to effectively improve mechanical performance, which is changing the type of binder and increasing the pressing pressure. Under the same pressing conditions, the order of compressive strength of PBX prepared by the three binders is FKM DS2603 > Viton A > PVAc. Moreover, the compressive strength of H-RDX-based PBX with FKM DS2603 can be increased by 33.7% compared with PVAc. When the pressing pressure is 200 MPa, the average compressive strength of H-RDX-based PBX with FKM DS2603 reaches 10.00 MPa, which can basically meet application requirements.

## 1. Introduction

With the continuous improvement of survival and safety requirements of munitions in modern warfare, there has been a huge demand for insensitive munitions (IM). Research suggests that one of the important potential method for reducing the vulnerability of explosive formulations is to improve the crystal quality of existing elementary explosives, reducing their sensitivity and improving their stability [1,2]. Due to the high detonation energy, low cost, and wide application of RDX, it is expected to obtain high-quality RDX (H-RDX) by improving the crystal quality [3,4,5]. The initial research topics focused on the improvement of RDX crystals and their product characterization [6,7,8,9]. Subsequently, the called I-RDX, RS-RDX, Grade A-RDX, and H-RDX were developed [10,11]. Additionally, the purity, density, mean size, shape, thermal, and hazard properties of H-RDX were also characterized [12,13,14]. Along with improvements in the quality of RDX crystal, the application research and performance evaluation of H-RDX has also been conducted over the years. The use of H-RDX is mainly concentrated in cast formulations and melt-cast formulations owing to its higher density, lower shock wave sensitivity, better thermal stability, and detonation performance [15,16,17,18,19]. Despite the application of H-RDX to pressed formulations coming later than to other kinds of formulations [20], RS-RDX can keep their reduced shock sensitivity feature in pressed formulations, even with some extra-granular pores in the bulk of the binder. Therefore, the effects of H-RDX on the safety and mechanical properties of pressed PBX were further studied.

Considering the coating of H-RDX is difficult due to its smooth surface. The H-RDX-based modeling powder was prepared by a water suspension granulation method [21] with three different binders. Moreover, the pressed PBX with a different binder was pressed in a stainless-steel mold under different compressive strengths. Finally, the formula with better mechanical properties was screened out, and impact sensitivity was tested to provide technical support for the large-scale application of H-RDX in pressed PBX.

## 2. Experimental Section

### 2.1. Materials

H-RDX particles (1.798 g•cm^−3^) and raw RDX (1.781 g•cm^−3^) with sizes ranging from 109 μm to 212 μm was procured from No.375 Factory of China North Industries Group Corporation (Liaoyang, Liaoning, China). PVAc with an average molecular weight of 40,000 g•mol^−1^ was supplied by the Sinopharm Chemical Reagent Co., Ltd. (Shanghai, China). DNT was purchased from No.804 Factory of China North Industries Group Corporation (Xian, China). The FKM DS2603 with fluorine content of 68% was provided by Shandong Huaxia Shenzhou New Material Co., Ltd. (Zibo, China). VITON A with a fluorine content of 66% was obtained from DuPont company. Ethyl acetate and stearic acid (both AR) were procured from Sinopharm Chemical Reagent Co., Ltd. (Shanghai, China). In the experiment, all reagents were used without further purification. Deionized water was used throughout the experiments.

### 2.2. Preparation of PBX

The H-RDX-based modeling powders were prepared by a water suspension granulation method [21] according to the formulation in Table 1. The binder was dissolved in ethyl acetate to form a binder solution (5%). The H-RDX (94.5 g) was placed in a 1000 mL three-necked flask, and then 300 mL water was added. This mixture was stirred at a speed of 250–350 rpm until uniformly dispersed. Keeping the inside flask temperature at 70 °C, the binder solution was added dropwise into the H-RDX suspension through a separating funnel over 20 min. Then, the mixture was heated to 90 °C. After about 30 min, SA was added and stirred for 5 min. When the temperature of the mixture fell to ambient temperature, the solid was filtered off, washed with water, and dried in a vacuum. The molding powders were, thus, obtained. Finally, the pressed PBX with different binders was pressed in a stainless-steel mold under different compressive strengths.

### 2.3. Experimental Techniques

The morphology of samples was measured by scanning electron microscope (SEM) and an optical microscope. The true density of samples was carried out at 298.15 K using an electronic weighing balance equipped density determination kit based on Archimedes principle. The samples were weighed both in air and in water; accordingly, density was calculated. The purity of H-RDX was analyzed by Agilent 1200 HPLC using a C18 chromatographic column and a methanol/acetonitrile/water mixture as the mobile phase, volume ratio of 28:12:60, and flow rate 1.5 mL/min. The injected volume was 5 μL. The UV detection wavelength was 240 nm. DSC analysis was carried out by a Mettler Toledo instrument from 50 to 600 °C, with a heating rate of 5, 10, 15, and 20 K•min^−1^ under nitrogen atmosphere (40 mL•min^−1^), and the mass of the powdery samples (H-RDX and PBXs) used was approximately 0.50 mg. The impact sensitivity was tested on a dropping hammer apparatus with a 5 kg drop weight according to GJB 772A-97 standard method 601.2 (National Military Standard of China) [22], and the results could be expressed by the drop height of 50% explosion probability (*H*_50_). The static mechanical tests were conducted with a universal testing machine at room temperature, with pressed cylindrical PBX for the compression test.

## 3. Results and Discussion

### 3.1. Morphological Analysis of H-RDX and Raw RDX

**Figure 1:**
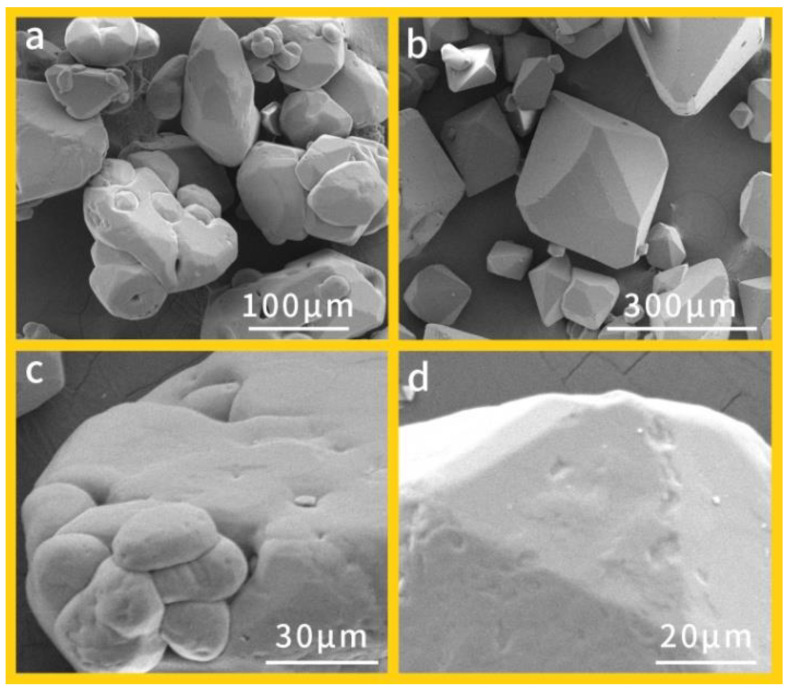
Apparent morphology images of different RDX crystals: (**a**) raw RDX (400×), (**b**) H-RDX (200×), (**c**) raw RDX (1500×), and (**d**) H-RDX (2000×).

In order to investigate the apparent morphology changes of RDX crystals, SEM and optical microscope were used to observe the surface and profile of H-RDX and raw RDX crystals. It can be observed from Figure 1a,c that raw RDX crystals have different shapes. In particular, the large raw RDX particles have many small crystals embedded or attached to the surface, resulting in obvious cracks and defects. However, a closer look at the H-RDX in Figure 1b,d points out that the crystal has a regular shape and a smooth surface. Compared with the raw RDX crystal, surface defects are significantly reduced. Table 2 shows the true densities and purities of H-RDX and raw RDX crystals. The H-RDX crystal has a much greater true density than raw RDX, which suggests that H-RDX has fewer voids. The purity of the H-RDX is higher than that of raw RDX, indicating H-RDX has fewer impurities. All of these results proved that the H-RDX crystal with less defects and inclusions is of a higher crystal quality.

### 3.2. Effects of H-RDX on the Safety Performance of PBX

The improvement of the quality of explosive crystals will cause changes in the safety performance of PBXs [4,7]. As the crystal shape of H-RDX is regular, the surface is smoother, the defect is less and the apparent density is higher so that the quality is significantly improved. Therefore, the accurate influence of H-RDX on the thermal safety and impact sensitivity of PBX was investigated.

#### 3.2.1. Effects of H-RDX on the Thermal Properties of PBX

**Figure 2:**
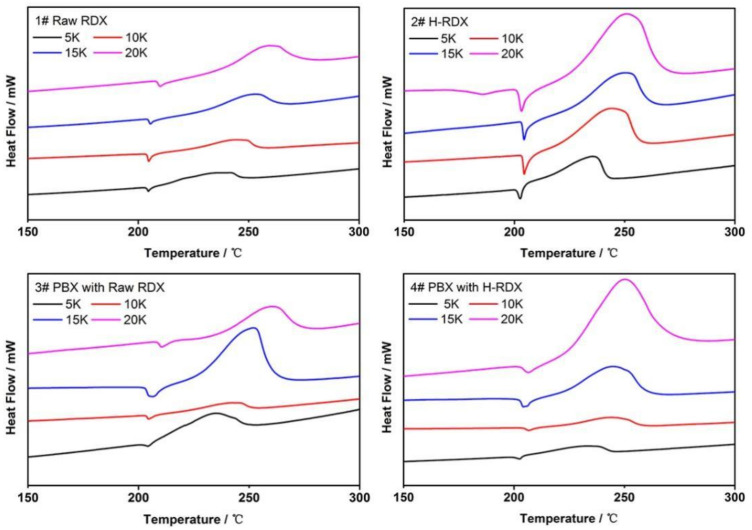
DSC curves of different RDX and PBX.

The DSC curves of the samples at different heating rates are shown in Figure 2. The thermal decomposition processes of H-RDX, raw RDX, and corresponding PBX are similar. As shown in Figure 2, there is an endothermic peak and an exothermic peak, and the endothermic peak near 204 °C (5 K•min^−1^) may be attributed to the melting of RDX, followed by an exothermic peak around 235 °C (5 K•min^−1^), which may correspond to the decomposition process of RDX. When the heating rate is 5 K•min^−1^ or 10 K•min^−1^, the thermal decomposition peak temperature of H-RDX is slightly higher than that of raw RDX, but it is not obvious.

In order to further compare the thermal properties of H-RDX, raw RDX, and corresponding PBX, the activation energy (*E*) of samples were studied by the Kissinger method and Flynn–Wall–Ozawa method, and the results are shown in Table 2. The equations of Kissinger (1) [23] and Ozawa (2) [24] are as follows.

(1) $$\ln\left(\frac{\beta_{i}}{T_{Pi}^{2}}\right)=\ln\frac{AR}{E}-\frac{E}{R}•\frac{1}{T_{Pi}}\;\;\;\;\;\;i=1,\;2,\;3,\;4$$

(2) $$lg\left(\frac{\beta_{i}}{T_{Pi}^{2}}\right)=\mathrm{lg}\frac{AE}{RG(\alpha )}-2.315-0.4567\frac{E}{RT_{Pi}}\;\;\;\;\;\;i=1,\;2,\;3,\;4$$

In those equations, *E* is the apparent activation energy (J•mol^−1^), *A* is the pre-referential constant (min^−1^), *β_i_* is the heating rate of the sample (K•min^−1^), *G*(*α*) is the reaction mechanism function, *T_pi_* is the peak temperature of thermal decomposition (K), and *R* is the ideal gas constant (8.314 J•mol^−1^•K^−1^).

It can be observed from Table 3 that the apparent activation energy calculated by the Kissinger method and the Flynn–Wall–Ozawa method are very close. The *E_K_* value of H-RDX is 63.64 kJ•mol^−1^ higher than that of raw RDX, which indicates that H-RDX has a high energy barrier, resulting in the thermal stability of the H-RDX being better than that of raw RDX. Furthermore, the *E_K_* of H-RDX-based PBX is 26.0% higher than that of raw RDX-based PBX, pointing out that applying H-RDX to pressed PBX can effectively improve its thermal stability, which is due to the fact that the thermal stability of the H-RDX crystal is better than raw RDX. Therefore, the quality of RDX crystal has a significant impact on the thermal safety of PBX. Overall, the use of H-RDX crystal results in an improvement of the thermal safety of PBX.

#### 3.2.2. Effects of H-RDX on the Impact Sensitivity of PBX

The impact sensitivity of raw RDX, H-RDX, raw RDX-based PBX, and H-RDX-based PBX was carried out using a 5 kg drop hammer. We performed 25 tests on each sample. *H*_50_ and Standard deviation (*S*) are shown in Table 4. The higher the *H*_50_, the lower the impact sensitivity. It can be observed from Table 4 that the *H*_50_ of H-RDX is 5 cm higher than that of raw RDX, indicating that the impact sensitivity of H-RDX is 23.7% lower than that of raw RDX. The main reasons related to this phenomenon have been studied. First, because the surface of H-RDX is smooth, it is easier to slide between particles and drop hammer when the particles were impacted, and the accumulated heat is lower. Second, the density of H-RDX is higher and the defects are less than that of raw RDX. It is known from previous research [25,26] that the fewer defects and the higher the crystal quality, the higher the impact sensitivity. Finally, *E_K_* of H-RDX is higher than that of raw RDX. Under the stimulation of external impact energy, the energy required for the H-RDX reaction will naturally be higher. Therefore, the *H*_50_ of H-RDX is higher and the impact sensitivity is lower.

Comparing 5 # and 6 #, it can be found that the *H_50_* of H-RDX-based PBX is 2.8 cm higher than that of raw RDX-based PBX, indicating that H-RDX can reduce the impact sensitivity of pressed PBX due to the fact that the impact sensitivity of H-RDX is lower than that of raw RDX. In addition, the analysis of 1 # and 5 # and 2 # and 6 # in Table 4 shows that the *H*_50_ of PBX increases more than one times after the RDX is coated, pointing out the impact sensitivity decreases greatly. According to the hot spot theory, the initiation of explosives is caused by the “hot spot” formed by local adiabatic compression. The binders are coated on the surface of the H-RDX crystal, which plays the role of a desensitizer. When the PBX is subjected to mechanical impact, the binders can first play a lubricating role, reducing the friction between PBX particles and reducing the generation of heat. Secondly, the endothermic melting of binders reduces the ignition energy of the supply hot spot, delaying the formation of a hot spot. Finally, the buffer energy absorption effect of the elastic-plastic material further reduces the probability of hot spot formation inside the H-RDX crystal.

In short, due to the smooth surface effect, dense effect, and higher *E_k_* of H-RDX, the impact sensitivity of H-RDX is lower than that of raw RDX, resulting in the impact sensitivity of H-RDX-based PBX being lower than that of raw RDX-based PBX.

### 3.3. Effects of H-RDX on the Mechanical Performance of Pressed PBX

The mechanical properties of pressed PBX are mainly related to the explosive crystals, the interface effect of binders, and explosive particles. The surface morphology of crystals will affect the adhesion between crystals and binders, thus resulting in changes in the mechanical properties of PBX. Since the surface of H-RDX is smoother and the morphology is more regular than that of raw RDX, the influence of H-RDX on the mechanical properties of pressed PBX was explored.

#### 3.3.1. Effect of Different RDX on Compressive Strength of Pressed PBX

**Figure 3:**
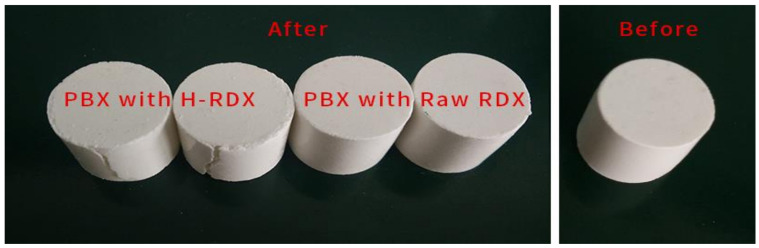
Comparison of PBX after compressive strength test.

**Figure 4:**
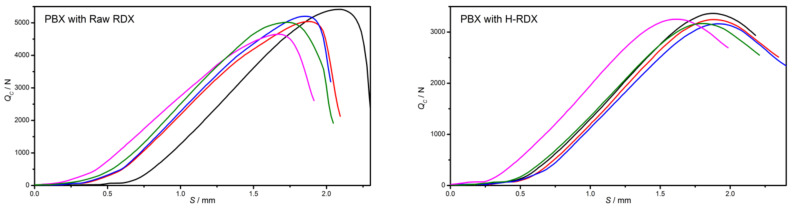
Load–displacement curves of PBX with different RDX.

The compressive strength (*S_c_*) of different RDX-based PBX prepared by 3 # and 4 # formulations with an applied pressure of 200 MPa was tested. The comparison of the samples before and after the test is shown in Figure 3. It can be observed that the surface cracks of H-RDX-based PBX are obvious after the test, while the surface of raw RDX is almost no cracks. A plot of the measured compressive stress as a function of the displacement is shown in Figure 4, and the test results are listed in Table 5. The compressive stress of H-RDX-based PBX is significantly lower than that of raw RDX-based PBX, and the compressive strength decreases by 36%. However, the fluctuation of compressive stress of H-RDX-based PBX is relatively small, due to its standard deviation and range being reduced by more than 60% compared with raw RDX-based PBX. In other words, the compressive strength consistency of H-RDX-based PBX is significantly better than that of raw RDX-based PBX.

In order to investigate the reason for the decline of compressive strength caused by H-RDX, we use the interfacial wetting theory [27,28] to deduce it. Since the surface roughness of H-RDX is different from that of raw RDX, the Young equation [29,30,31] is applied to the rough surface system. Combined with the Young equation and Wenzel equation [32], we can obtain Equation (3):

*r* = cos*θ*′/cos*θ* > 1 (3)

where *r* is a rough factor, *θ*′ is the apparent contact angle of solid–liquid phase (°), and *θ* is the solid–liquid contact angle (°).

Equation (3) shows that the cos*θ*′ of a rough surface is always greater than that of a smooth surface. The apparent morphology analysis of RDX proves that the surface of H-RDX is smoother than that of raw RDX; thus, we have the following.

cos*θ*′ _RDX_ > cos*θ*′ _H-RDX_ (4)

Reference [18] shows that the liquid surface tension of PVAc, FKM DS2603, and VITON A is always greater than 0, for the use of the same binder in PBX. Combined with Equation (4) and Young equation, we obtain the following:

*W_a_*_RDX_ > *W_a_*_H-RDX_ (5)

where *W_a_* is the adhesion work (mJ•m^−2^). According to Equation (5), the adhesion work between H-RDX with smooth surface and binder is smaller than that of raw RDX and binder, which means that the interface effect between H-RDX crystal and binder is weaker; thus, the compressive strength of pressed H-RDX-based PBX is lower.

On the other hand, the irregularity of the surface, such as uneven peak valley or loose pore structure, is conducive to the filling of the adhesive. After curing, the adhesive and the surface are bitten and fixed. In the process of coating RDX with a water suspension granulation method, the binder wets or spreads on the irregular surface of RDX crystal, which plays the role of filling peak valleys and gaps and makes the surface of RDX crystal form a large area of close combination with the binder, which result in the physical “interlocking block” [33]. Therefore, the raw RDX-based PBX with an irregular surface has better compressive strength properties than the H-RDX-based PBX.

In summary, the surface of H-RDX is smoother, and the compressive strength of H-RDX-based PBX is lower. However, due to the regular surface shape of H-RDX, the stress in each direction is uniform. Thus, the compressive strength of H-RDX-based PBX is better consistent.

#### 3.3.2. Effect of Pressing Intensity on the Compressive Strength of Pressed H-RDX-Based PBX

The pressing intensity of the PBX will directly affect the density and porosity of the PBX, thereby affecting the mechanical properties of the pressed PBX. Therefore, the mechanical properties of H-RDX-based PBX prepared by 4 # molding powder under holding time of 8 s, pressing pressure of 42 kN (100 MPa), 52 kN (125 MPa), 62 kN (150 MPa), and 83 kN (200 MPa) were studied, as listed in Table 6.

It can be observed from Table 6 that the compressive strength of pressed H-RDX-based PBX increases with an increase in pressing intensity in the range of 100 MPa~200 MPa. According to the statistical theory of micro-crack brittle damage [34], there are inevitably initial defects such as microcracks in the preparation process of PBX. When the pressing intensity becomes higher, the density of the PBX is higher, yet the porosity of the PBX is lower; thus, PBX has fewer and smaller initial microcracks or other defects. Under the stimulation of external pressure, the smaller initial microcracks expand steadily and slowly, thereby reducing the crack’s growth rate. Therefore, pressed PBX with fewer and smaller initial microcracks needs a greater external load to achieve the critical damage degree, which means that it can withstand greater compressive strength. In summary, the increase in pressing intensity can improve the compressive strength of PBX by reducing porosity, initial cracks, and other defects.

#### 3.3.3. Effect of Different Binders on the Compressive Strength of Pressed H-RDX-Based PBX

**Figure 5:**
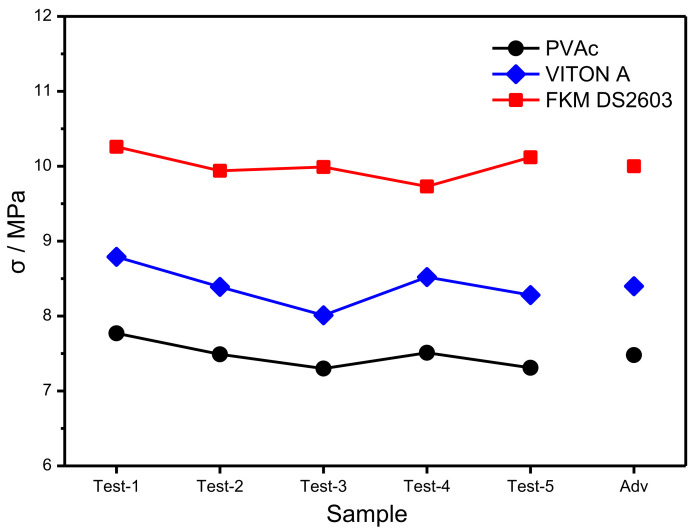
Compressive strength of PBX with different binders.

Thanks to the compressive strength of pressed H-RDX-based PBX decreased significantly, the feasibility of improving the compressive mechanical properties of PBX was explored by changing the binder. Firstly, H-RDX-based molding powder was prepared according to the formula of 4 # (PVAc), 6 # (FKM DS2603), and 7 # (VITON A), and then PBX was pressed with an applied pressure of 200 MPa. Finally, their compressive strength was tested, and the results are shown in Figure 5.

It can be observed from Figure 5 that the compressive strength of pressed H-RDX-based PBX prepared with the three binders is in the order of FKM DS2603 > VITON A > PVAc. The largest average compressive strength of pressed H-RDX-based PBX prepared with FKM DS2603 binder reaches 10.00 MPa, which is 33.7% higher than the smallest. Meanwhile, the compressive strength of raw RDX-based PBX (3 #) pressed with an applied pressure of 150 MPa is 9.97 MPa, proving that the FKM DS2603/H-RDX formula (6 #) can meet the use requirements.

#### 3.3.4. The Discussion of the Interfacial Reinforcement Effects between H-RDX and Binders

Compared with PVAc/H-RDX composites, the compressive strength of fluororubber/H-RDX composites was significantly improved, which was believed to originate from the interface reinforcement effect of fluororubber on H-RDX. It can be attributed to the following mechanisms.

According to the adsorption theory [27,28,29,30,31,32], the binder is adsorbed on the surface by wetting, and the movement of macromolecules or molecular chains forms a diffusion interface area. Furthermore, the molecules undergo physical adsorption or chemical reaction, forming a bond that crosses the secondary or main chemical valence of the interface. First, the -F group in fluororubber (FKM DS2603 or VITON A) molecule has high electronegativity and, thus, produces strong dipole–dipole interactions with the -NO_2_ group on the surface of H-RDX crystal. Although the CH_3_COO- group in PVAc can also produce dipole–dipole interactions with the -NO2 group on the surface of the H-RDX crystal, the volume of the CH_3_COO- group is larger and electronegativity is weaker than that of the -F group; thus, the dipole–dipole interaction between CH_3_COO- group and -NO_2_ group is smaller. Additionally, for binders with the same weight, the number of −F groups of fluororubber is 156% more than that of CH_3_COO- groups of PVAc, according to the molecular force summation formula of Fowkes [27,28,29], and the total force between fluororubber and H-RDX crystal is also larger. At the same time, the fluorine content of FKM DS2603 is 2% higher than that of VITON A thus, the total dipole–dipole interaction between FKM DS2603 and H-RDX is larger, which results in the increase in intermolecular interaction and enhances the compressive strength of H-RDX-based PBX.

Diffusion theory [27,28,29,30,31,32] believes that the flexibility of the molecular chain increases, the side group reduces, and the crosslinking degree raises, and these are beneficial to molecular diffusion, resulting in bonding strength increases. The CH_3_COO- group of PVAc is suspended outside the main chain, resulting in an increase in the spatial steric hindrance of the diffusion interface, which inhibits the spread of the PVAc molecular chain on the surface of the H-RDX crystal. Nevertheless, the space volume of the -CF_3_ group on the fluororubber molecular chain is smaller than that of the CH_3_COO- group; thus, its space steric hindrance is small, which is convenient for the diffusion of fluororubber molecular chain and molecular chain on the surface of H-RDX crystal to achieve greater bonding strength.

In addition, the H-RDX molecule contains the -NO_2_ group, which is an electron donor and belongs to the Lewis base. However, the F atom in the fluororubber molecule has a strong ability to attract electrons, resulting in a positive charge around the C atom of the main chain, becoming a proton donor and belonging to Lewis acid. Moreover, the CH_3_COO- group in PVAc can provide an electron pair, which belongs to the Lewis base. Consequently, according to the acid-base interaction theory [28,32], -NO_2_ groups of H-RDX are easier to form coordination bonds with fluororubber molecules and increase the molecular interaction at the interface.

It is revealed that the interface reinforcement effect of fluororubber on H-RDX by the analysis of adsorption theory, diffusion theory and acid-base interaction theory. The essential reason is that the fluororubber molecular chain is easier to wet and diffuse on the surface of H-RDX crystal, forming coordination bonds and obtaining stronger dipole–dipole interaction. Hence, the compressive strength of the pressed H-RDX-based PBX can be improved by the use of fluororubber, which enhances molecular interaction on the interface.

## 4. Conclusions

The different RDX-based PBX was prepared by pressing modeling powder, which was coated with a binder by a water suspension granulation method. Furthermore, the effects of H-RDX on the safety and mechanical properties of pressed PBX were fully studied. The surface of the H-RDX crystal is smoother, and the shape is more regular than that of raw RDX. The defects are significantly reduced, the activation energy of H-RDX is 63.64 kJ•mol^−1^ higher than that of raw RDX, and *H*_50_ is 5 cm higher than that of raw RDX. Thus, H-RDX has good thermal stability, low impact sensitivity, and high intrinsic safety. Moreover, the *E_K_* of H-RDX-based PBX is 26.0% higher than that of raw RDX-based PBX, and the *H*_50_ is 2.8 cm higher than that of raw RDX-based PBX, which point out that the application of H-RDX to PBX can effectively improve its thermal stability and decrease the impact sensitivity in safety performance. However, the compressive strength of pressed H-RDX-based PBX is 36% lower than that of pressed raw RDX-based PBX, showing that H-RDX results in the deterioration of the compressive strength of pressed PBX in mechanical performance. Fortunately, this work provides a potential method to improve mechanical performance, which is changing the type of binder and increasing the pressing pressure. When the pressing pressure is 200 MPa, the average compressive strength of H-RDX-based PBX with FKM DS2603 reaches 10.00 MPa, which can basically meet application requirements.

## Figures and Tables

**Table 1:** Formulations of PBX with different RDX %.

Sample	RDX	H-RDX	DNT	PVAc	FKM DS2603	VITON A	SA
1 #	100	0	0	0	0	0	0
2 #	0	100	0	0	0	0	0
3 #	94.5	0	3	2	0	0	0.5
4 #	0	94.5	3	2	0	0	0.5
5 #	94.5	0	0	0	5	0	0.5
6 #	0	94.5	0	0	5	0	0.5
7 #	0	94.5	0	0	0	5	0.5

**Table 2:** The true density and purity of different RDX crystals.

Sample	True Density/g·cm^−3^	Purity/%
1 #	1.781	99.14
2 #	1.798	99.91

**Table 3:** *T_p_* of samples at different heating rates and calculated value of *E_a_*.

Sample	*T_p_*/° C				*E_K_*/ kJ•mol^−1^	*E_O_*/ kJ•mol^−1^
	5 K•min^−1^	10 K•min^−1^	15 K•min^−1^	20 K•min^−1^		
1 #	235.41	243.97	252.98	261.03	111.87	114.62
2 #	235.71	244.31	250.03	251.66	175.51	175.07
3 #	234.75	242.5	252.22	260.77	108.10	111.03
4 #	232.35	243.93	245.03	252.53	146.06	147.04

Note: Subscript K and O data obtained by Kissinger’s method and Flynn–Wall–Ozawa’s method from *T_p_*.

**Table 4:** Impact sensitivity of different RDX and PBX.

Sample	1 # (RDX)	2 # (H-RDX)	5 #	6 #
*H*_50_/cm	21.1	26.1	56.2	59.0
*S*/cm	0.27	0.08	0.08	0.08

Note: *S* is the Standard deviation.

**Table 5:** Compressive strength of PBX with different RDX.

Sample	*ρ*/g·cm^−3^	*Q_C_*/N	Average *Q_C_*/N	*S*/N	Range/N	Average *S_C_*/MPa
3 #	1.65	5412.90	5063.73	280.78	763.50	11.69
	1.66	5039.92				
	1.66	5201.03				
	1.66	4649.40				
	1.66	5015.40				
4 #	1.69	3365.78	3237.94	82.74	204.01	7.47
	1.68	3244.07				
	1.69	3161.77				
	1.69	3251.08				
	1.68	3167.02				

Note: *Q_C_* is the compressive stress of PBX. *S_C_* is the compressive strength of PBX. *S* is the Standard deviation.

**Table 6:** Compressive strength of PBX with different pressing pressure.

Stress/kN	*P*/MPa	Pressed Density/g·cm^−3^	Porosity/%	*S_C_*/MPa
42	100	1.63	8.8	5.24
52	125	1.65	7.7	5.86
62	150	1.66	7.1	6.39
83	200	1.68	6.0	7.47

## Data Availability

The data underlying this article will be shared on reasonable request from the corresponding author.

## Abbreviations and Acronyms

RDX	1,3,5-Trinitroperhydro-1,3,5-triazine
H-RDX	High-quality RDX
I-RDX	Insensitive RDX
RS-RDX	Reduced sensitivity RDX
PBX	Plastic Bonded Explosive
FKM DS2603	Fluororubber DS2603
VITON A	Fluororubber VITON A
PVAc	Polyvinyl acetate
DNT	2,4-Dinitrotoluene
SA	Stearic acid
*H*_50_	The drop height of 50% explosion probability
*E*	The activation energy
G(α)	Reaction mechanism function
*S*_c_	Compressive strength
*Q*_c_	Compressive stress
*S*	Standard deviation
*W*_a_	Adhesion work
*r*	Rough factor

## References

[1] Powell I.J. (2016). Insensitive Munitions—Design Principles and Technology Developments. Propellants Explos. Pyrotech..

[2] Anniyappan M., Talawar M.B., Sinha R.K., Murthy K.P.S. (2020). Review on Advanced Energetic Materials for Insensitive Munition Formulations. Combust. Explos. Shock Waves.

[3] Johansen Ø.H., Kristiansen J.D., Gjersøe R., Berg A., Halvorsen T., Smith K.-T., Nevstad G.O. (2008). RDX and HMX with Reduced Sensitivity Towards Shock Initiation–RS-RDX and RS-HMX. Propellants Explos. Pyrotech..

[4] Spyckerelle C., Eck G., Sjöberg P., Amnéus A.-M. (2008). Reduced Sensitivity RDX Obtained From Bachmann RDX. Propellants Explos. Pyrotech..

[5] Freche A., Aviles J., Donnio L., Spyckerelle C. Insensitive RDX (I-RDX). Proceedings of the Insensitive Munitions and Energetic Materials Technology Symposium.

[6] van Ham N.H.A., van der Steen A.C., Meulenbrugge J.J. Less Sensitive Explosives. Proceedings of the AGARD Conference.

[7] van der Steen A.C., Duvalois W., Hordijk A.C. Crystal Quality and Less Sensitive Explosives. Proceedings of the Insensitive Munitions Technology Symposium.

[8] Rui J.-H., Zhao X. (2013). A Study on Preparation and Properties of High Density RDX Crystal. Acta Armamentarii.

[9] Wang Y., Li X., Chen S., Ma X., Yu Z., Jin S., Li L., Chen Y. (2017). Preparation and Characterization of Cyclotrimethylenetrinitramine (RDX) with Reduced Sensitivity. Materials.

[10] Spyckerelle C., Donnio L., Aviles J., Freche A. Analytical Characterization of Insensitive RDX. Proceedings of the 32nd International Annual Conference of ICT.

[11] Kim J.W., Kim J.K., Kim H.S., Koo K.K. (2009). Characterization of liquid inclusion of RDX crystals with a cooling crystallization. Cryst. Growth Des..

[12] Zhao X., Rui J.-H., Feng S.-S. (2011). Recrystallization Method for Preparation of Spherical RDX. Trans. Beijing Inst. Technol..

[13] Chen G., Xia M., Lei W., Wang F., Gong X. (2013). A study of the solvent effect on the morphology of RDX crystal by molecular modeling method. J. Mol. Model..

[14] Herrmann M., Förter-Barth U., Bohn M.A., Borne L. (2017). Aging of Standard and Insensitive RDX Crystals Investigated by Means of X-Ray Diffraction. Propellants Explos. Pyrotech..

[15] van der Steen A.C., Skjold E. RDX Particle Shape and the Sensitivity of PBXs. Proceedings of the Joint Government/Industry Symposium on Insensitive Munitions Technology.

[16] van der Heijden A., Bouma R., van der Steen A. (2004). Physicochemical Parameters of Nitramines Influencing Shock Sensitivity. Propellants Explos. Pyrotech..

[17] Doherty R.M., Watt D.S. (2008). Relationship Between RDX Properties and Sensitivity. Propellants Explos. Pyrotech..

[18] Wang D.-X., Chen S.-S., Li Y.-Y., Yang J.-Y., Wei T.-Y., Jin S.-H. (2013). An Investigation into the Effects of Additives on Crystal Characteristics and Impact Sensitivity of RDX. J. Energetic Mater..

[19] Li T., Hua C., Li Q. (2013). Shock Sensitivity of Pressed RDX-Based Plastic Bonded Explosives under Short-Duration and High-Pressure Impact Tests. Propellants Explos. Pyrotech..

[20] Borne L., Mory J., Schlesser F. (2008). Reduced Sensitivity RDX (RS-RDX) in Pressed Formulations: Respective Effects of Intra-Granular Pores, Extra-Granular Pores and Pore Sizes. Propellants Explos. Pyrotech..

[21] Yang Z., Lin C., Gong F., Zeng C., Zhang J., Huang F. (2019). Effects of Crystal Quality and Morphology on the Mechanical Performance of LLM-105 Based PBXs. Propellants Explos. Pyrotech..

[22] China Ordnance Industry Standardization Research Institute (1997). National Military Standard of China. Experimental Methods of Sensitivity and Safety.

[23] Kissinger H.E. (1957). Reaction kinetics in differential thermal analysis. Anal. Chem..

[24] Ozawa T.A. (1965). New method of analyzing thermogravimetric data. Bull. Chem. Soc. Jpn..

[25] Armstrong R., Ammon H., Elban W., Tsai D. (2002). Investigation of hot spot characteristics in energetic crystals. Thermochim. Acta.

[26] Huang X., Qiao Z., Dai X., Zhang K., Li M., Pei G., Wen Y. (2019). Effects of different types of defects on ignition mechanisms in shocked β-cyclotetramethylene tetranitramine crystals: A molecular dynamics study based on ReaxFF-lg force field. J. Appl. Phys..

[27] Bhushan B. (1999). Principles and Applications of Tribology.

[28] Israelachvili J.N. (1992). Intermolecular and Surface Forces.

[29] Adamson A.W., Klerer J. (1977). Physical Chemistry of Surfaces. J. Electrochem. Soc..

[30] Zhang X.-L., Liu M.-E., Liu W.-Z., Deng J.-R. (2016). Synthesis and Interfacial Adhesion Interaction of Borate Ester Bonding Agents Used for HTPB Propellants. Propellants Explos. Pyrotech..

[31] Schrader E.M. (1995). Young-Duprerevisited. Langmuir.

[32] Wenzel R.N. (1936). Resistance of solid surfaces to wetting by water. Ind. Eng. Chem..

[33] Jin X., Heepe L., Strueben J., Adelung R., Gorb S.N., Staubitz A. (2014). Challenges and Solutions for Joining Polymer Materials. Macromol. Rapid Commun..

[34] Dienes J.K., Middleditch J., Kershner J.D., Zuo Q.K., Starobin A.J. Progress in statistical crack mechanics: An approach to initiation. Proceedings of the 12th International Symposium on Detonation.

