# Correction: Chen et al. The Effect of High-Quality RDX on the Safety and Mechanical Properties of Pressed PBX. *Materials* 2022, *15*, 1185

**DOI:** 10.3390/ma15062315

**Published:** 2022-03-21

**Authors:** Shixiong Chen, Hua Qian, Bingxin Liu, Feiyang Xu, Jiuhou Rui, Dabin Liu

**Affiliations:** 1 School of Chemical Engineering, Nanjing University of Science and Technology, Nanjing 210094, China; shixiong_chen@126.com (S.C.); qianhua@njust.edu.cn (H.Q.); 18252501712@163.com (B.L.); iem_liu@163.com (F.X.); 2 State Key Laboratory of Explosion Science and Technology, Beijing Institute of Technology, Beijing 100081, China

**Error in Figure**

In the original publication [1], there was a mistake in **Figure 1** as published. **The number 10 in Figure 1a needs to be replaced with 100**. The corrected **Figure 1** appears below.

The authors apologize for any inconvenience caused and state that the scientific conclusions are unaffected. The original publication has also been updated.

## Figures and Tables

**Figure 1:**
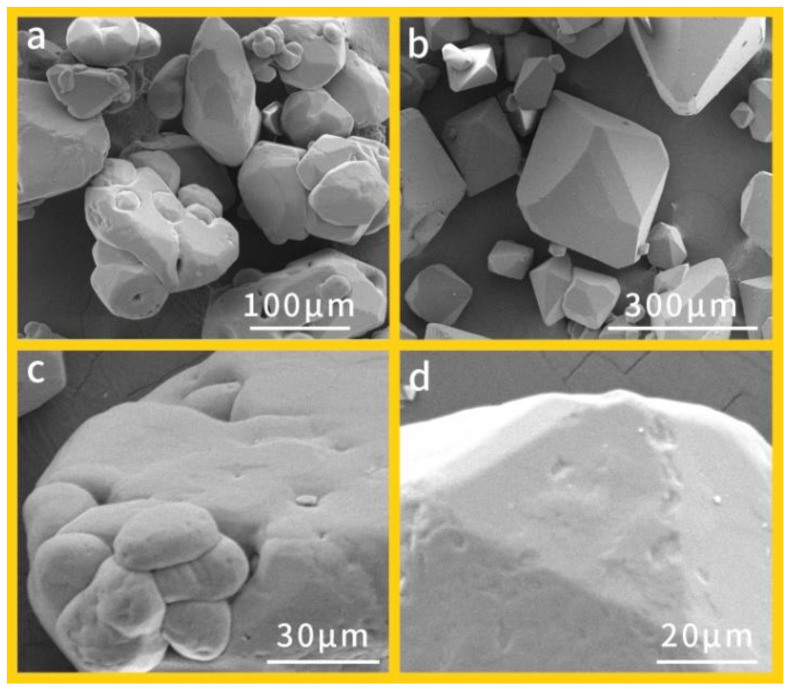
Apparent morphology images of different RDX crystals: (**a**) raw RDX (400×), (**b**) H-RDX (200×), (**c**) raw RDX (1500×), and (**d**) H-RDX (2000×).
